# Identification and Experimental Validation of Triosephosphate Isomerase 1 as a Functional Biomarker of SHetA2 Sensitivity in Ovarian Cancer

**DOI:** 10.3390/cells15030267

**Published:** 2026-01-30

**Authors:** Laura F. Mortan, Zitha Redempta Isingizwe, Doris Mangiaracina Benbrook

**Affiliations:** 1 Gynecologic Oncology Section, Stephenson Cancer Center, University of Oklahoma Health Campus, Oklahoma City, OK 73104, USA; laura-mortan@ou.edu (L.F.M.); zitha-isingizwe@ou.edu (Z.R.I.); 2 Pathology Department, University of Oklahoma Health Campus, Oklahoma City, OK 73104, USA; 3 Obstetrics and Gynecology Department, College of Medicine, University of Oklahoma Health Campus, Oklahoma City, OK 73104, USA

**Keywords:** high grade serous ovarian carcinoma, ascites, SHetA2, olaparib, triosephosphate isomerase 1, biomarker, glycolysis, pentose phosphate pathway, TCA cycle, spheroids

## Abstract

Background: Our objective was to identify and validate proteins that predict which patients with ovarian cancer will respond to SHetA2, an investigational drug in a phase 1 trial for patients with advanced or recurrent solid tumors (clinicaltrials.gov: NCT04928508). Methods: Cells were cultured from ascites from nine consented patients under an institutional review board-approved protocol. SHetA2 or olaparib sensitivities were determined using metabolic viability assays in ascites-derived cultures or ovarian cancer cell lines. Expression of four SHetA2 target proteins and sixteen proteins previously identified in an ovarian cancer mouse model were measured using microcapillary electrophoresis. Triosephosphate isomerase 1 (TPI1) was modulated by siRNA or lentivirus vector-mediated overexpression. Metabolites were measured using mass spectrometry. Results: TPI1 was elevated in SHetA2-sensitive compared to SHetA2-resistant ascites-derived cultures (two-way ANOVA q-value = 0.0003). The majority of (5/9) cultures were olaparib-resistant and SHetA2-sensitive. TPI1 was higher in olaparib-resistant cultures (two-way ANOVA q-value = 0.0003). Reduction in or overexpression of TPI1 reduced or increased SHetA2 potency, respectively, in two ovarian cancer cell lines (*t*-tests; *p* < 0.05). SHetA2 reduced the metabolites in glycolysis downstream of TPI1, the tricarboxylic acid cycle and oxidative pentose phosphate pathway. Conclusions: TPI1 is a candidate functional biomarker of SHetA2 sensitivity in ovarian cancer.

## 1. Introduction

Ovarian cancer is one of the leading causes of cancer deaths among women [1,2]. Owing to the lack of specific symptoms and early detection strategies, more than half of the estimated 20,000 patients with ovarian cancer are diagnosed at an advanced stage. Current standard of care for advanced ovarian cancer involves surgical debulking followed by six cycles of platinum-based doublet chemotherapy, with the potential to include targeted therapy [3,4]. Targeted therapies consist of antiangiogenic agents and poly (ADP-ribose) polymerase (PARP) inhibitors, olaparib, rucaparib, and niraparib [5]. Most patients with stage III or IV ovarian cancer will undergo a period of remission before having a recurrence of their cancer, resulting in a five-year survival rate of only 25% [1,5,6]. Research into the biology of ovarian cancer provides insight into the mechanisms of this high rate of recurrence.

Ovarian cancer is a heterogenous disease consisting of multiple histologies arising from different organ sites [7,8]. High-grade serous ovarian carcinoma (HGSOC), the most common and deadly histology, can arise directly from surface epithelium or inclusion cysts of the ovary or from serous tubal intraepithelial carcinoma lesions of the fallopian tube that migrate to the ovary. As HGSOC progresses, cancer cells that are shed from the ovarian tumors metastasize primarily to the omentum and then to other sites and organs throughout the peritoneal cavity [9,10]. In advanced cancer cases, blockage of lymphatic drainage by the metastatic tumors and increased capillary permeability driven by cancer production of angiogenic factors causes the buildup of a fluid called ascites in the peritoneal cavity [11]. The ascites fluid from patients with HGSOC consists of cancer cells and non-cancer cells (mesothelial, fibroblast, immune, endothelial) and other factors (cytokines, chemokines, proteases, cell-free DNA, exosomes) and is highly heterogenous between patients [11,12]. While some of the factors in the ascites tumor microenvironment can foster cancer survival and metastases, other factors are tumor-suppressive [12,13]. Our comparison of ascites from patients with primary versus recurrent HGSOC revealed a more tumor-suppressive immune microenvironment in the recurrent ascites specimens [14].

The cancer cells in ascites are present as single cells, spheroids, or mixtures of single cells and spheroids. HGSOC cells with stem cell-like properties (CSCs), such as the capacity for self-renewal, can seed spheroids and form metastatic lesions and appear to be responsible for chemotherapy resistance and cancer recurrence [15,16]. A major factor behind HGSOC CSC ability to survive hostile ascites tumor microenvironments and chemotherapy is their metabolic plasticity (capacity to reprogram their metabolism, e.g., switching between oxidative phosphorylation and glycolysis) [17,18,19]. These cancer cells present in the ascites of patients with HGSOC represent a source of recurrence that could be targeted with secondary prevention strategies.

After patients with HGSOC respond to standard-of-care treatment, maintenance therapy is typically administered as a secondary prevention strategy to delay cancer recurrence. The current drugs used in maintenance therapy are anti-angiogenic or PARP inhibitor-targeted drugs, such as olaparib [20,21]. However, these maintenance therapies are limited by the development of unacceptable toxicities and recurrence in most patients [22,23]. Thus, there is a significant demand for novel therapeutic approaches in patients who experience recurrence and exhibit resistance to existing treatments.

SHetA2 is a promising new investigational drug that has documented activity against HGSOC without toxicity in preclinical models [24]. Extensive preclinical data demonstrated that SHetA2 did not cause skin irritation, teratogenicity, cardiotoxicity or general toxicity at doses up to 50-fold above those shown to inhibit tumor growth [25]. A phase 1 clinical trial of oral SHetA2 capsules in patients with advanced or recurrent solid tumors (clinicaltrials.gov: NCT04928508) has shown no dose-limiting toxicities and preliminary anti-tumor activity. SHetA2 works by disrupting the chaperone functions of heat shock protein 70 kDa (HSP70) family members, glucose-regulated protein 78/Grp78, heat shock cognate 70/hsc70 and mortalin [26]. These proteins serve as molecular chaperones by facilitating the folding, degradation, and/or intracellular localization of client proteins and protein complexes. HSP70s are elevated in cancer cells where they bind elevated client oncoproteins, including cyclin D1 or p53, and protect the cell from the deleterious effects of the client protein overexpression. SHetA2 treatment of cancer cells causes the release of client oncoproteins, causing their degradation or re-localization within the cell [24,27]. Downstream consequences of the mortalin complex disruption by SHetA2 include mitochondrial damage, inhibition of oxidative phosphorylation and mitochondria-selective autophagy (mitophagy) that contributes to cell death in ovarian cancer cells [28]. In contrast, non-cancerous ovarian or fallopian tube epithelial cells can counteract the mitochondrial effects of SHetA2 through mitochondrial fusion and low-level autophagy, whereas ovarian cancer cells are defective in these survival mechanisms [28]. While the mechanism of SHetA2 is becoming elucidated, there remains a need to identify and validate proteins within this mechanism that could be used as biomarkers to predict which patients will respond to SHetA2 therapy.

The objective of this study was to identify and validate biomarkers of SHetA2 sensitivity. To direct our study, we utilized data from our previous orthotopic mouse models of ovarian cancer. In one model, we demonstrated that oral gavage with SHetA2 significantly reduced the establishment of peritoneal tumors in mice injected with an HGSOC cell line [24]. In the other model, we identified genes that were differentially expressed in peritoneal tumors compared to cells floating in ascites fluid [29]. We hypothesized that proteins encoded by the differentially expressed genes and SHetA2 drug targets (cyclin D1, Grp78, hsc70 and mortalin) are involved in how SHetA2 prevents tumor establishment and will predict SHetA2 sensitivity. To test this hypothesis, we collected and evaluated ascites-derived cells from patients with HGSOC that recurred after treatment standard of care chemotherapy or a second-line experimental antibody drug conjugate. Olaparib was used as control to verify the specificity of our method and the biomarkers identified. Higher levels of aldehyde dehydrogenase 1 family member A3 (ALDH1A3), triosephosphate isomerase 1 (TPI1), and tropomyosin 1 (TPM1) were associated with SHetA2 sensitivity, while higher levels of mitogen-activated protein kinase 1(MAPK1), TPI1 and TPM1 were associated with olaparib resistance. The majority of cultures were resistant to olaparib while being sensitive to SHetA2. Validation testing by altering specific protein expression when combined with metabolomic analysis verified TPI1 is a candidate biomarker of SHetA2 sensitivity in ovarian cancer.

## 2. Materials and Methods

### 2.1. Patient Eligibility and Consent

This research was conducted on clinically annotated specimens from patients aged 18 and above, who had presumed or confirmed cancer and ascites confirmed by a clinical imaging procedure. Patients fulfilling the criteria were recruited at the Stephenson Cancer Center (SCC) or the University of Oklahoma (OU) Health Campus, Oklahoma City, OK, USA. Informed consent was obtained from all subjects, allowing for the examination of ascites fluid in a study conducted according to the guidelines of the Declaration of Helsinki. Ascites fluid was collected prospectively during paracentesis or surgery according to the OU Health Campus IRB-approved protocol IRB #15770. Patient demographic information was obtained from the electronic medical record review (IRB #7328). Only specimens from patients with recurrent HGSOC were included in this study.

### 2.2. Specimen Processing

Ascites specimens were immediately transported from the clinic or the operating room to a cell culture hood in a lab where they were aliquoted into sterile 50 mL centrifuge tubes. Following centrifugation at 250× *g* for ~5 min, the cell-free supernatant and the resuspend pellet were aliquoted, flash-frozen in liquid nitrogen and stored at −80 °C. The ascites cells and spheroids were aliquoted by volume directly into U-bottom ultra-low-attachment 96-well plates for viability assays. Tissue Protein Extraction Reagent (T-PER Fisher Scientific, Cat# 78510, Hampton, NH, USA) was used to extract protein from the cells within the ascites, and BCA (Pierce BCA, Cat# 23225, Thermo Scientific, Waltham, MA, USA) was used to quantify the protein concentration.

### 2.3. Measurement of Protein Levels

In addition to four SHetA2 target proteins, proteins encoded by genes previously identified to be differentially expressed in tumor versus ascites in our previous orthotopic model of HGSOC [29] were evaluated. Sixteen of the seventy differentially expressed genes were chosen for evaluation based on their results not being driven by extreme outliers. Levels of specific proteins (ALDH1A3; aldolase A, fructose-bisphosphate/ALDOA; caveolin 1, caveolae protein, 22 kDa/CAV1; CD44 molecule/CD44; Cyclin D1; GABA(A) receptor-associated protein/GABARAP; Grp78; hsc70; integrin, beta 1/ITGB1; integrin, beta 3/ITGB3; lactate dehydrogenase A/LDHA; lysyl oxidase/LOX; MAPK1/mortalin; serpin peptidase inhibitor, clade E, member 1/PAI1; phosphoglycerate kinase 1/PGK1; pyruvate kinase muscle/PKM1; TPI1; tropomyocin/TPM1) were evaluated using the Protein Simple Jess Microcapillary Electrophoresis System (Minneapolis, MN, USA). The 12-230 kDa separation module (Bio-Techne R&D Systems #SM-W001, Minneapolis, MN, USA) was used to separate equal protein concentrations of whole protein lysates. Samples stored at −80 °C were thawed over ice and mixed with Simple Western Sample Buffer (0.1×) (Bio-Techne R&D Systems #PS-ST01EZ, Minneapolis, MN, USA) to a total of 1.5 µg total protein, and denatured. Samples, Total Protein Reagent (Bio-Techne R&D Systems #DM-TP01, Minneapolis, MN, USA), primary (Supplemental Table S1) and secondary antibodies (Bio-Techne R&D Systems #DM-001 (Rabbit) Minneapolis, MN, USA), Chemiluminescent Substrate (Bio-Techne R&D Systems, Minneapolis, MN, USA), and Replex Reagent (Bio-Techne R&D Systems #RP-001, Minneapolis, MN, USA) were dispensed into designated wells in the Jess Simple Western plate. The plate was then centrifuged at 1000× *g* for 5 min, after which it was placed in the Protein Simple Jess instrument with the capillary cartridge. The Simple Western Compass program was used to identify peaks recognized by the primary and secondary antibodies matching the appropriate molecular weights for the specific proteins. Numbers from the area under the curve (AUC) for the identified peaks for the specific proteins derived by the Compass Software were then transferred to GraphPad Prism version 10.2.1 for further analysis.

### 2.4. Drug Dilutions

Stocks of SHetA2 (synthesized by Dr. K. Darrell Berlin, Oklahoma State University, Stillwater, OK, USA) were dissolved to 10 mM in DMSO. Olaparib (Cat# 04402, LOT # 2599933, LKT Laboratories, St. Paul, MN, USA) stocks were dissolved to 20 mM in DMSO. Drug stocks were kept at −20 °C until used. The drugs were diluted with the same media used to culture cells to achieve 4× the final desired concentrations.

### 2.5. Ascites-Derived Cell Culture Viability Assays

Ascites-derived cell cultures were plated in low-attachment 96-well plates in 50 μL/well of ascites fluid to allow the cells to grow as spheroids. All drug or negative control treatments were administered in 50 μL/well high-glucose Dulbecco’s Modified Eagle’s Medium containing 10% fetal bovine serum (FBS) and 1% antibiotic antimycotic (DMEM). Single-drug treatments were administered by adding 25 μL of each drug at 4× concentration into the respective wells and diluting 25 μL DMEM containing the same volume of DMSO used to dissolve the drug. Drug combinations were prepared by adding 25 μL of 4× concentrations in an orthogonal matrix. Viability of ascites cultures was measured by a CellTiter-Glo 3D Cell Viability Assay (Promega Cat# G9683, Madison, WI, USA). Each treatment was performed in triplicate on each of at least 3 replicate 96-well plates. The viability data for the triplicate plates were normalized to the untreated control and then plotted against the drug doses, and the half-maximal inhibitory concentrations (IC_50′_s) were derived using Prism 10.0 (GraphPad, San Diego, CA, USA).

### 2.6. Human Ovarian Cancer Cell Lines

The human ovarian cancer cell lines ES2/Luciferase-2A-GFP (RRID:CVCL_E8YF), ES-2 (RRID:CVCL_3509), or MES-OV (RRID:CVCL_CZ92) were cultured in DMEM high-glucose media containing 10% fetal bovine serum, 1% antibiotic and antimycotic. Cultures underwent authentication and mycoplasma testing by IDEXX (Columbia, MO, USA).

### 2.7. Effect of Inhibition of SHetA2 Biomarkers Expression on Metabolic Viability

Genetic inhibition was performed using siRNA and Lipofectamine RNAiMAX reagent (Cat# 13778075, Thermo Fisher Scientific, Waltham, MA, USA). Silencer Select Negative Control Reagent (4390843, Cat #4390843, Thermo Fisher Scientific, Waltham, MA, USA) was used as the negative control for each siRNA. Validated human siRNA against TPI1(4390824- s14339, Cat# 43820) was used for silencing. Cell viability in the transfected cultures −/+ a range of SHetA2 concentrations was then measured in standard 96-well cell culture plates by Cell Titer 96 Non-Radioactive Cell Proliferation Assay (Promega #G4100, Madison, WI, USA). The siRNA transfection and subsequent viability assay was performed 3 times in triplicate.

### 2.8. Effect of Overexpression of SHetA2 Biomarkers on Metabolic Viability

A custom overexpression lentiviral vector for TPI1 was designed using Vector Builder software (Chicago, IL, USA) and purchased from VectorBuilder Inc. (Chicago, IL, USA). The ES-2 and MESOV cell lines were transfected and selected for puromycin expression, which was confirmed using Protein Simple Jess evaluation of TPI1 protein expression. Cell Viability was then measured by a Cell Titer 96 Non-Radioactive Cell Proliferation Assay (Promega #G4100, Madison, WI, USA) as described in the above paragraph. Each transfection and subsequent viability assay was performed 3 times in triplicate.

### 2.9. Treatment of Cells for Metabolomics Analysis

The human ES-2/Luciferase-2A-GFP ovarian cancer cell line was cultured until 90% confluence and then treated in triplicate with SHetA2 at 5 µM or DMSO vehicle control for either 5 min or 6 h before replacing the media with 2000 mg/L (11.11 mM) of U-^13^C-labeled glucose +/− SHetA2. The cells were labeled with U-^13^C-labeled glucose. Each group was run in triplicate alongside pooled samples for quality control.

### 2.10. Extraction of Polar Metabolites

Cells were washed with PBS and HPLC-grade water sequentially, then quenched with ice-cold LC-MS-grade methanol. Quenched cells were scraped, collected into Eppendorf tubes, and dried using a SpeedVac. Metabolites are extracted by adding 1 mL of ice-cold methanol/acetonitrile/water (2:2:1, *v*/*v*/*v*) to each sample, followed by vortexing, flash-freezing in liquid nitrogen, and sonication for 10 min at 25 °C, repeated for 3 consecutive cycles. Extracts were incubated at −20 °C for at least 1 h and centrifuged at 14,000× *g* for 10 min at 4 °C. Supernatants were collected into new Eppendorf tubes and dried using a SpeedVac. Protein content from the residual cell pellets was quantified using a BCA assay to normalize metabolite extraction volumes. Dried metabolite extracts were reconstituted by adding 1 µL of acetonitrile/water (2:1, *v*/*v*) per 2.5 µg of protein. Samples were vortexed, followed by sonication for 10 min at 25 °C, repeated twice. Finally, extracts were centrifuged at 14,000× *g* and for 10 min at 4 °C, and supernatants were transferred to LC vials and stored at −80 °C until LC-MS analysis.

### 2.11. LC/MS Analysis of Metabolites

Ultra-high-performance liquid chromatography coupled with mass spectrometry (UHPLC/MS) analyses were conducted using a Vanquish Flex UHPLC system (Thermo Scientific, Waltham, MA, USA), interfaced with a Orbitrap ID-X Mass Spectrometer (Thermo Scientific, Waltham, MA, USA). For the separation of polar metabolites, a iHILIC-(P) Classic HILIC column (100 × 2.1 mm, 5 µm) (HILICON AB, Umea, Swedon) with a iHILIC-(P) Classic guard column (20 × 2.1 mm, 5 µm) (HILICON AB, Umea, Swedon) was utilized. The mobile-phase solvents consisted of solvent A = 20 mM ammonium bicarbonate, 2.5 µM medronic acid, 0.1% ammonium hydroxide in 95:5 water/acetonitrile and solvent B = 95:5 acetonitrile/water. The column compartment temperature was maintained at 45 °C, and metabolites were eluted using a linear gradient at a flow rate of 250 μL/min as follows: 0–1 min, 90% B; 12 min, 35% B; 12.5–14.5 min, 25% B; 15 min, back to 90% B. Data was acquired in positive and negative ion modes. The LC/MS data were then processed and analyzed using X^13^CMS version 1.4, CompoundDiscoverer version 3.3.200, Skyline version 25.1.0.142, and AccuCor version 0.3.1 [30,31,32].

### 2.12. Metabolomics Data Analysis

Metabolomics data were reviewed, and compounds with >20% coefficient of variation or without measurable peaks in >20% of all samples were eliminated. Data analysis was performed using MetaboAnalyst version 6.0 (Wishart Research Group, University of Alberta, Edmonton, AB, Canada) [33]. Data were scaled using Log10 transformation and auto-scaling (mean-centered and divided by the standard deviation of each variable). Human metabolome database (HMDB) IDs were inserted and matched to standardized compound labels within the pathway library. The exact matches of each metabolite from the test dataset to the pathway library proceeded to analysis. The compounds without matches did not proceed to pathway analysis.

### 2.13. Statistical Analysis

Normality and log-normality tests were run on each protein expression data set to determine the distribution. The data was determined to be lognormally distributed by the Shapiro–Wilk test, then an unpaired lognormal Welch’s *t*-test was conducted to compare the protein expression levels in drug-sensitive compared to drug-resistant groups. SHetA2 dose–response curves were compared by the extra sum of squares F test, which compares the independent fits of each curve with a global fit that shares the bottom, top, IC_50_ and Hill Slope to determine if these parameters are significantly different between the two curves and provides a *p*-value to determine if the null hypothesis that the curves are the same can be rejected. All statistical analyses were performed using Prism 10.0 (GraphPad, San Diego, CA, USA).

## 3. Results

### 3.1. Sensitivity of SHetA2 in Ascites Samples

Cells and spheroids aliquoted from ascites specimens collected from patients with recurrent HGSOC were cultured under conditions that maintained spheroids for 24 h and then treated with various SHetA2 concentrations for 72 h before measuring their metabolic viability. The patients had recurred after being treated with standard of care chemotherapy consisting of carboplatin alone or in combination with paclitaxel and bevaxizumab, or an experimental drug raludotatug deruxtecan (RM 720), a CDH6-targeting antibody–drug conjugate with a DNA topoisomerase I inhibitor. Plots of the metabolic viability versus drug dose were used to derive the potencies (half-maximal inhibitory concentrations [IC_50′_s]) of SHetA2 for each ascites-derived culture (Table 1). For the first seven cultures of HGSOC collected and evaluated (ASC4, ASC7, ASC12, ASC 21, ASC23, ASC24 and ASC25), a subset (ASC12, ASC 21, ASC23 and ASC24) were classified as SHetA2-sensitive because they were inhibited at SHetA2 doses below the highest tested (20 µM) with SHetA2 potencies ranging from 1.3 to 18.3 µM. Another subset of the cultures was classified as SHetA2-resistant (ASC4, ASC7, and ASC25) because they were not inhibited at SHetA2 doses below 20 µM and had dose–response lines with slopes that were not significantly different from zero and therefore undefined potencies (ASC4, ASC7, and ASC25).

### 3.2. Identification of Predictive Biomarkers of SHetA2 Sensitivity

**Figure 1:**
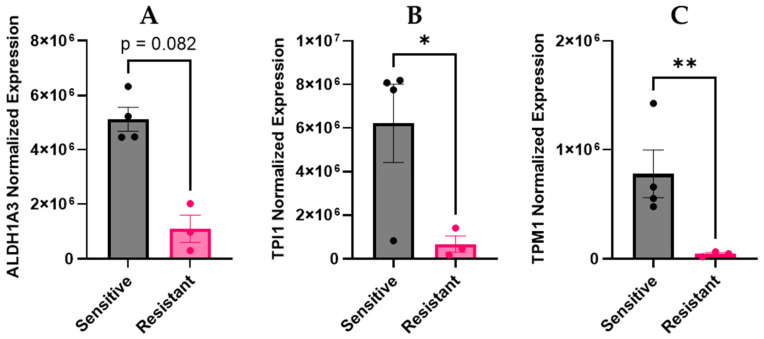
Identification of biomarkers of SHetA2 sensitivity from patient ascites. SHetA2-sensitive (ASC12, ASC 21, ASC23, ASC24) and SHetA2-resistant (ASC4, ASC7 and ASC25) ascites-derived culture groups described in Table 1 were compared for levels of individual proteins: (**A**) ALDH1A3, (**B**) TPI1, (**C**) TPM1. Each dot represents an individual ascites culture. (* = *p*-value < 0.05, ** *p*-value < 0.005.)

Comparison of the SHetA2-sensitive versus SHetA2-resistant ascites-derived culture groups provided an opportunity to identify biomarkers of SHetA2 drug sensitivity. To begin this exploration, we evaluated four SHetA2-target proteins and 16 proteins encoded by genes that we previously identified to be differentially expressed in ascites cells compared to solid tumor specimens of an orthotopic ovarian cancer mouse model [29]. We hypothesized that the mechanism of our previous observation of SHetA2 prevention of peritoneal ovarian cancer establishment in mice could involve interference with these proteins [24]. Comparison of the normalized protein expression levels between the SHetA2-sensitive and SHetA2-resistant groups revealed that two of the twenty proteins, TPI1 and TPM1, were significantly higher in the SHetA2-sensitive ascites-derived cultures (Figure 1). In addition, there was a trend of higher ALDH1A3 expression in SHetA2-sensitive samples (Figure 1). After correcting for multiple comparisons using a two-way ANOVA mixed-effects analysis with correction by the two-stage linear step-up procedure of Benjamini, Krieger and Yekutieli, the TPI1 and ALDH1A3 differences between sensitive and resistant cultures were statistically significant q values of 0.0003 and 0.005, respectively, while the TPM1 q value was nonsignificant. There were no significant differences in the *p* values among the other 17 of the 20 proteins evaluated (Supplementary Table S1).

### 3.3. Specificity of SHetA2 Sensitivity and Biomarkers Profiles

**Figure 2:**
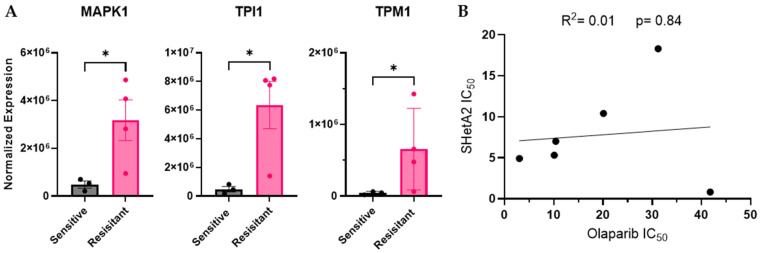
Olaparib validation of SHetA2 predictive biomarkers. (**A**) Comparison of MAPK1 and TPI1 expression in olaparib-sensitive (ASC7, ASC12 and ASC25) versus olaparib-resistant (ASC4, ASC21, ASC23, ASC24) ascites-derived cultures. Unpaired lognormal Welch’s *t*-test (*p*-values * < 0.05). (**B**) Correlation of SHetA2 and olaparib IC_50′_s in cultures sensitive to both drugs: ASC 12, ASC 21, ASC 23, ASC 24, ASC 32, and ASC 62. In each graph, each dot represents an individual ascites culture.

To evaluate the specificity of our strategy for identifying SHetA2 sensitivity biomarkers, we used the identical approach on the same ascites-derived cultures to identify olaparib sensitivity biomarkers. In a study by Amuzu et al. [34], human HGSOC cell lines with potencies of 7 µM or greater were considered olaparib-resistant, and those potencies of <7µM were considered olaparib-sensitive. Furthermore, the multiple-dosing steady-state maximal concentration of olaparib measured in the blood is 7.6 µg/mL or 17.5 µM, which documents that this dose is clinically achievable. Therefore, our ascites-derived cultures exhibiting olaparib potencies below 7 µM were categorized as olaparib-sensitive, while those with potencies above 7 µM were categorized as olaparib-resistant. Compared to SHetA2, olaparib exhibited a unique profile of proteins associated with ascites sensitivity and resistance to treatment. Of the 20 screened proteins evaluated, MAPK1, TPI1 and TPM1 were significantly higher in the olaparib-resistant group than in the olaparib-sensitive group (Figure 2A). Our two-way ANOVA analysis with corrected *p* values for multiple comparisons confirmed that TPI1 had significantly higher protein levels in the resistant compared to the sensitive cultures with a q value of 0.0001, while MAPK1 exhibited a trend for a higher protein level with a q value of 0.06. There were no significant differences in the other 17 of the 20 proteins evaluated (Supplementary Table S1). Among the samples that were sufficiently sensitive to both SHetA2 and olaparib to derive IC_50_ values_,_ there was no correlation between potencies for these two drugs (Figure 2B).

We then compared the profiles of ascites-derived cell culture sensitivities to SHetA2 versus olaparib. Additional cultures (ASC32 and ASC62) were included in further analysis as they were prospectively collected. Of the nine ascites cultures, six were sensitive to SHetA2, three were sensitive to olaparib, and one was resistant to both (Table 2). The majority (five) of the cultures were resistant to olaparib while being sensitive to SHetA2, suggesting that SHetA2 could be effective as an alternative maintenance therapy in patients with olaparib-resistant HGSOC.

### 3.4. Validation of Biomarker Protein Expression Levels with SHetA2 Potency

**Figure 3:**
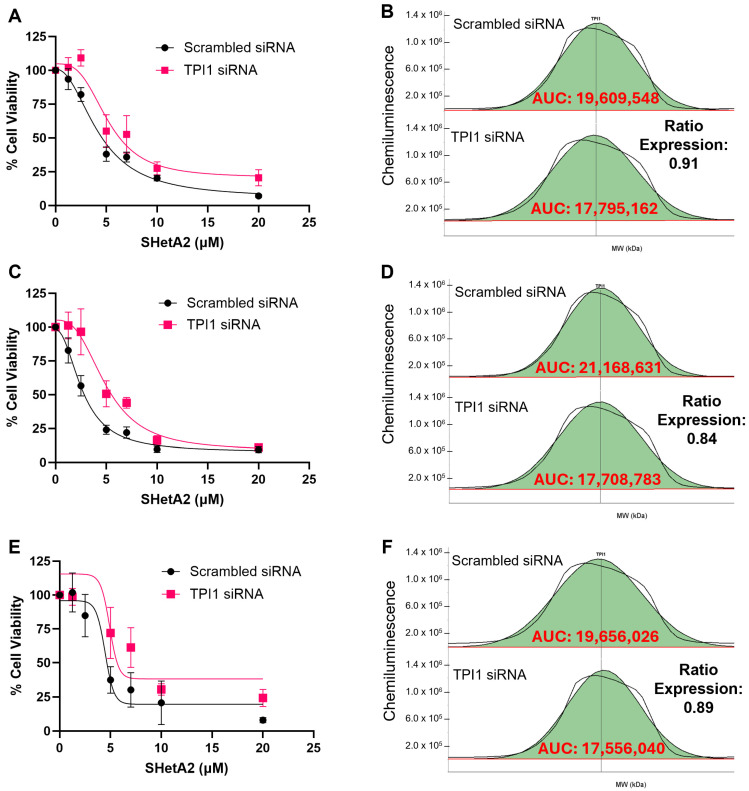
Comparison of SHetA2 dose–response effects on ES-2 cell viability in cultures transfected with scrambled siRNA control compared to TPI siRNA. (**A**,**B**) Replicate 1. (**B**,**C**) Replicate 2. (**D**,**E**) Replicate 3. (**A**,**C**,**E**) Dose–response curves. (**D**–**F**) Comparison of area under the curve (AUC) of histograms detecting TPI1 protein Jess Protein Simple microcapillary electrophoresis runs of whole cell protein extracts isolated from cultures transfected with scrambled siRNA compared to TPI siRNA.

**Figure 4:**
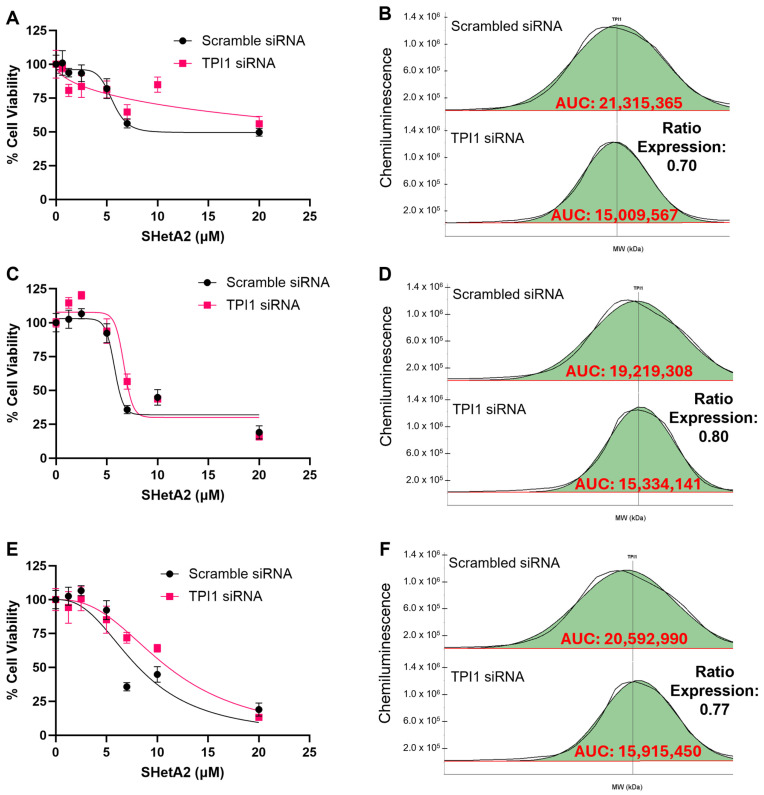
Comparison of SHetA2 dose–response effects on MESOV cell viability in cultures transfected with scrambled siRNA control compared to TPI siRNA. (**A**,**B**) Replicate 1. (**B**,**C**) Replicate 2. (**D**,**E**) Replicate 3. (**A**,**C**,**E**) Dose–response curves. (**D**–**F**) Comparison of area under the curve (AUC) of histograms detecting TPI1 protein Jess Protein Simple microcapillary electrophoresis runs of whole cell protein extracts isolated from cultures transfected with scrambled siRNA compared to TPI siRNA.

**Figure 5:**
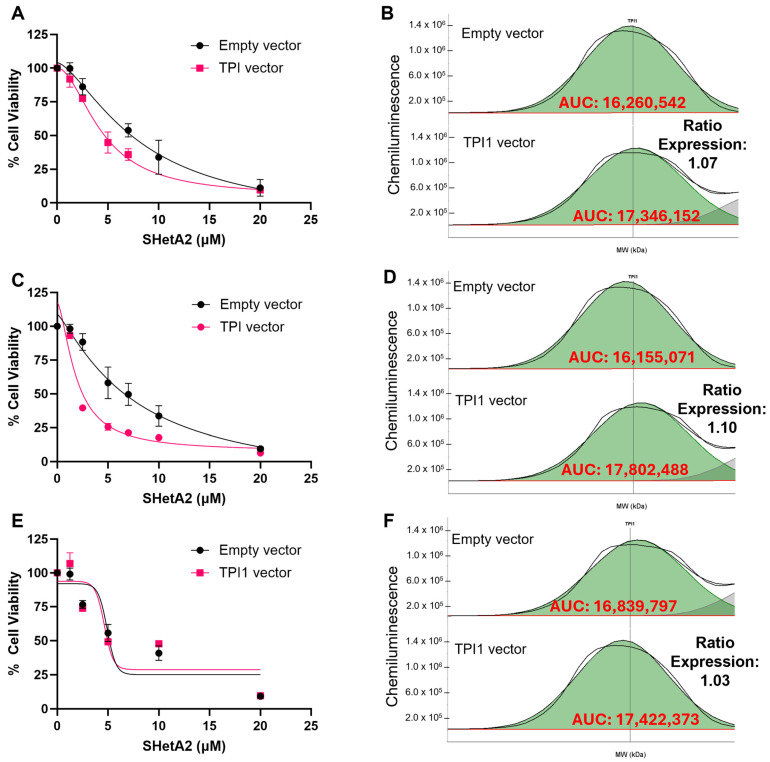
Comparison of SHetA2 dose–response effects on ES-2 cell viability in cultures transfected with empty vector control compared to TPI vector. (**A**,**B**) Replicate 1. (**B**,**C**) Replicate 2. (**D**,**E**) Replicate 3. (**A**,**C**,E) Dose–response curves. (**D**–**F**) Comparison of area under the curve (AUC) of histograms detecting TPI1 protein Jess Protein Simple microcapillary electrophoresis runs of whole cell protein extracts isolated from cultures transfected with empty vector controls compared to TPI vector.

**Figure 6:**
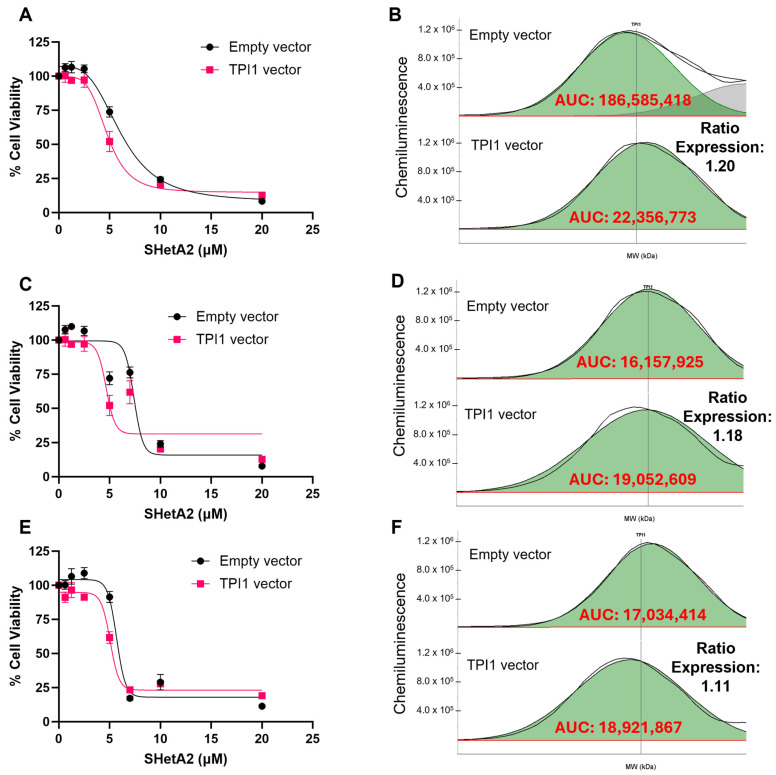
Comparison of SHetA2 dose–response effects on MESOV cell viability in cultures transfected with empty vector control compared to TPI vector. (**A**,**B**) Replicate 1. (**B**,**C**) Replicate 2. (**D**,**E**) Replicate 3. (**A**,**C**,**E**) Dose–response curves. (**D**–**F**) Comparison of area under the curve (AUC) of histograms detecting TPI1 protein Jess Protein Simple microcapillary electrophoresis runs of whole cell protein extracts isolated from cultures transfected with empty vector controls compared to TPI vector.

To determine whether the TPI1 candidate biomarker of SHetA2 sensitivity merely correlates with or is functionally involved in the drug mechanism of action, we tested whether alternating its expression by siRNA silencing or viral vector overexpression significantly altered SHetA2 potency or efficacy. Because ascites cultures proved technically difficult to transfect, we utilized the ES-2 and MESOV human HGSOC cell lines. The ES-2 cell line was originally misclassified as being derived from clear cell ovarian cancer; however, its genomic characterization is similar to that of HGSOC due to *BRAF* and *TP53* gene mutations [35]. SHetA2 dose–response curves were generated and used to derive the potency and efficacy of SHetA2 for each genetic intervention and compared with that of the appropriate negative control. The results exhibited consistent patterns of siRNA TPI1 reduction interfering with SHetA2 cytotoxicity in comparison with the scrambled control (Figure 3 and Figure 4), and TPI1 protein overexpression enhancing SHetA2 cytotoxicity in comparison to the empty vector control (Figure 5 and Figure 6). Comparison of the dose–response curve fits using the extra sum-of-squares F test demonstrated that curves corresponding to the control versus siRNA or overexpression intervention were significantly different, except in one instance in the ES-2 cell line in which TP overexpression of only 1.03-fold was achieved (Table 3).

### 3.5. Validation of SHetA2 Interference with Metabolomics Downstream of TPI1

**Figure 7:**
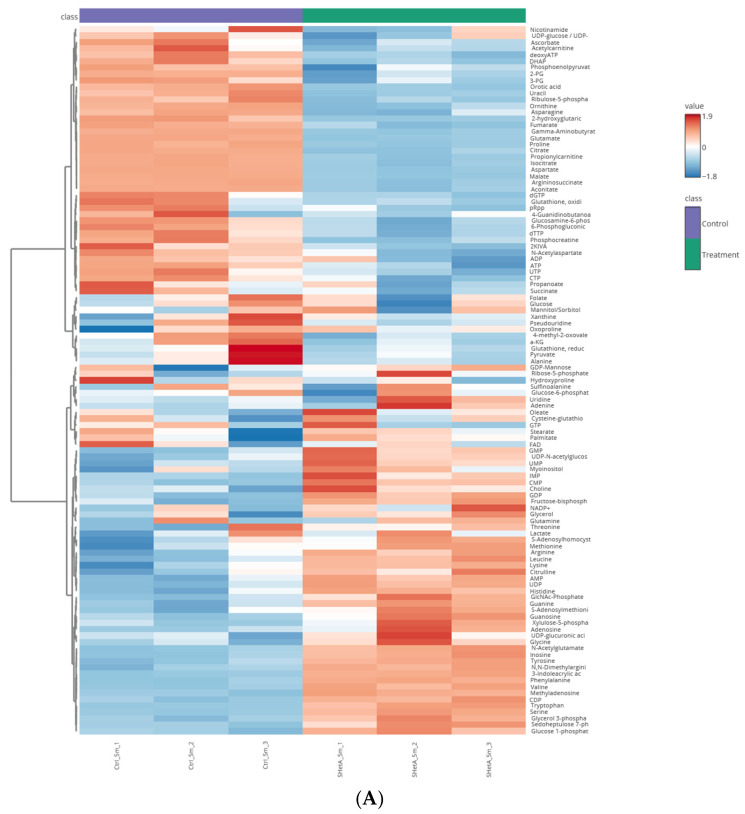

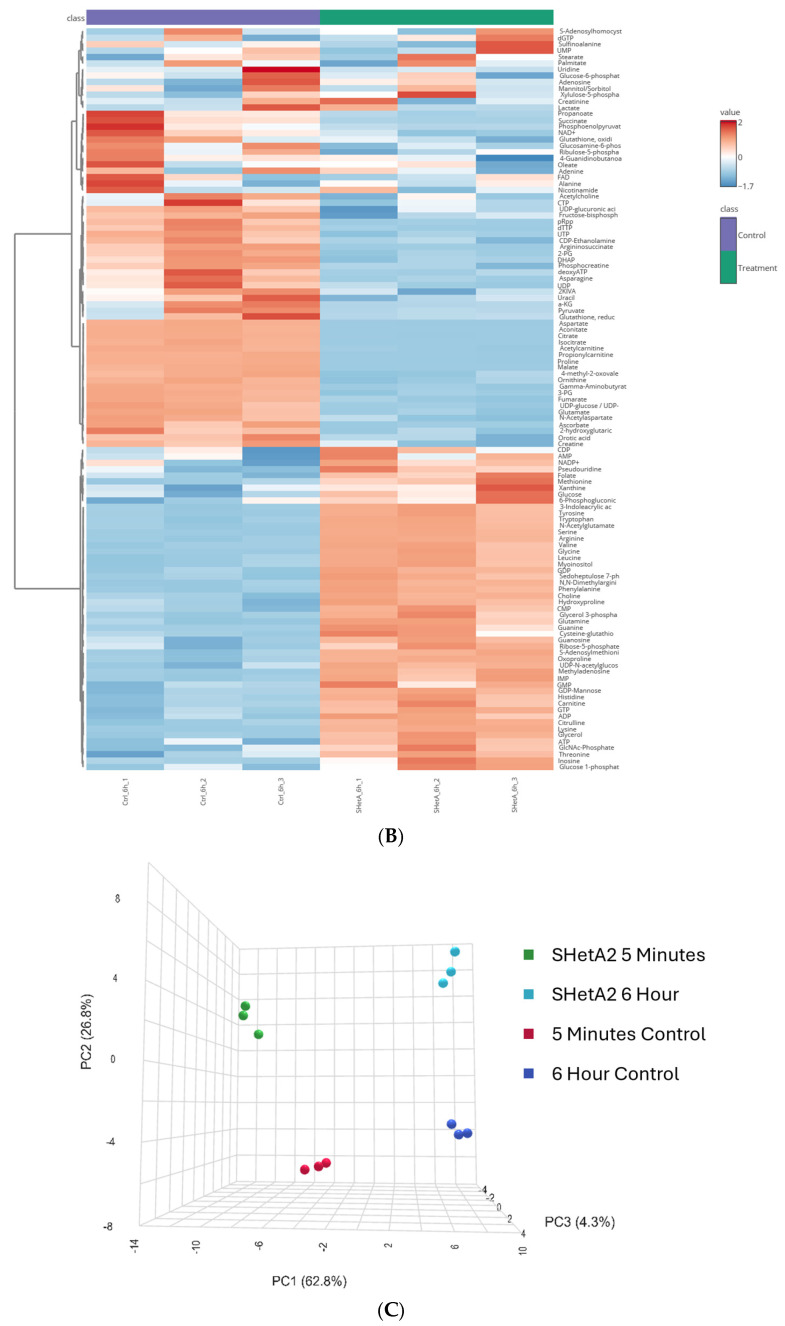
Metabolomic effects of SHetA2 in the ES-2 ovarian cancer cell line. Heat maps of metabolites (**A**) after 5 min and (**B**) 6 h of SHetA2 treatment, and (**C**) Principal Component Analysis.

Because TPI1 plays a central role in glycolysis, we validated its functional role in the SHetA2 mechanism of action by evaluating the effects of SHetA2 on metabolomic profiles in the ES-2 cells using mass spectrometry (Supplementary Table S2). The cell cultures were treated with 5 µM SHetA2 for 5 min prior to U-^13^C-glucose −/+ SHetA2 to investigate the glycolytic and pentose phosphate pathway (PPP) intermediates (Figure 7A), and with SHetA2 for 6 h prior to U-^13^C-glucose −/+ SHetA2 to investigate citric acid (TCA) cycle intermediates and connected metabolites (Figure 7B). Principal Component Analysis demonstrated distinct clustering between the SHetA2 treated and control samples (Figure 7C).

**Figure 8:**
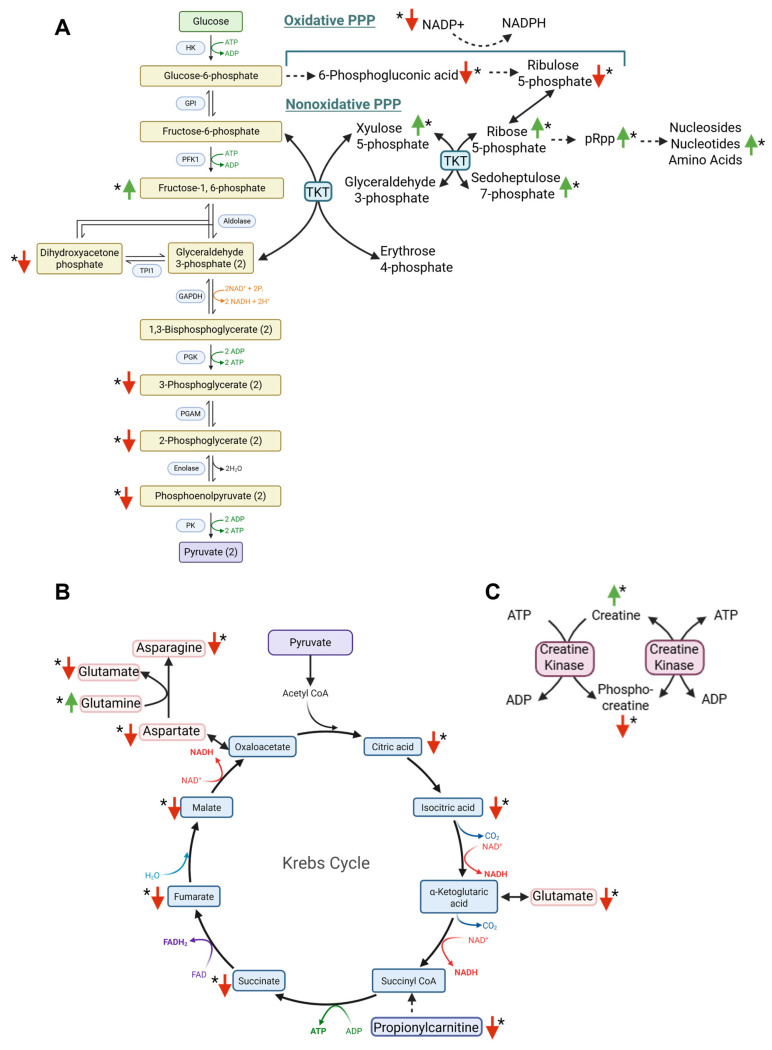
Illustrations of SHetA2 metabolomic effects in ovarian cancer cells. (**A**) Significant effects of SHetA2 after 5 min of treatment. (**B**) Significant effects of SHetA2 after 6 h of treatment. (**C**) Significant effects of SHetA2 on phosphocreatine were observed at both 5 min and 6 h of treatment. Asterisks indicate *p* < 0.05, straight red arrows indicate decreased metabolites, straight green arrows indicate increased metabolites. Solid black arrows indicate direct reactions. Dotted arrows indicate multiple steps. Curved arrows of various colors indicate coenzymes used in reactions.

The 5 min metabolomic study demonstrated that SHetA2 blocks glycolysis at TPI1, leading to reduced levels of downstream metabolites (dihyroxyacetone phosphate, 3-phosphoglycerate, 2-phosphoglycerate and phosphoenylpyurvate) and increased levels of the upstream metabolite (fructose-6-phosphate) (Figure 8A). The nonoxidative PPP was also increased by SHetA2 as indicated by increased xyulose 5-phosphate, ribose 5-phosphate, sedoheptulose 7-phosphate, phosphoribosyl pyrophosphate (pRpp) which is a known precursor of nucleosides and nucleotides, and which could explain the elevated AMP, ADP, ATP, dATP, CMP, CDP, CTP, GMP, GDP, GTP, and IMP. However, the only deoxynucleotide measured dTTP was decreased. pRpp is also a precursor of histidine, which was also elevated in SHetA2-treated cultures. In contrast, oxidative PPP, which generates NADPH, was inhibited by SHetA2 as indicated by decreased 6-phosphogluconic acid and ribulose 5-phosphate and in association with decreased NADP+.

The 6 h metabolomic study demonstrated that SHetA2 blocked the TCA cycle, reduced TCA metabolites and the associated amino acids aspartate, asparagine and glutamate (Figure 5B). The aspartate-malate shuttle, which shuttles electrons from glycolysis to the mitochondria, appears to be decreased based on the reduced levels of oxaloacetate, malate, aspartate and glutamate metabolites. Propionyl-carnitine, which can be used to generate ATP when it is converted to propionyl-CoA and then into succinyl-CoA to feed the TCA cycle, was also reduced (Figure 8B). The creatine kinase pathway, which is also used to generate ATP in the cell by converting phosphocreatine to creatine, also appears to be inhibited by SHetA2, as indicated by significantly reduced levels of phosphocreatine at both 5 min and 6 h of SHetA2 treatment (Figure 8C). Additional alterations of amino acid levels by SHetA2 in ovarian cancer cells include a significant elevation of arginine, glycine, histidine, leucine, lysine, methionine, phenylalanine, serine, threonine, tyrosine, tryptophan and valine and reduction in proline.

In addition, we conducted an enrichment pathway analysis which confirmed these findings and identified additional pathways that were affected by SHetA2 treatment (Supplementary Tables S3 and S4).

## 4. Discussion

The potential success of clinical trials can be enhanced by screening patients to identify those most likely to benefit from the intervention being tested. Immunohistochemical analysis of tumors is often used to predict drug sensitivity; however, it is not always feasible to obtain recurrent tumor specimens for predictive biomarker analysis in clinical trials in the recurrent setting. In contrast to invasive surgical techniques required to remove solid tumors, the ascites fluid that commonly accumulates in patients with recurrent HGSOC is more easily collected through a procedure called paracentesis that drains the ascites through a needle in the clinic or through a vacuum-based suction procedure in the operating room before surgery begins. HGSOC ascites represents an accessible rich source of information about the disease. Multiple studies have reported the promise of using ascites specimens to predict how patients with cancer will respond to drugs [36,37,38,39,40,41]. In this study, we utilized ascites from patients with HGSOC who recurred after standard-of-care chemotherapy or an experimental antibody drug conjugate to identify biomarkers of sensitivity to our investigational new drug, SHetA2.

The recurrent HGSOC cultures in our study exhibited a range of sensitivities to SHetA2, which gave us the opportunity to categorize the cultures into SHetA2-sensitive and SHetA2-resistant based on the potency (IC_50_ value) derived for each culture. Candidate biomarkers were identified by comparing differentially expressed proteins between the two categories using a targeted approach. We took advantage of a gene set previously identified to be differentially expressed between tumor and ascites and verified to be differentially expressed at the protein level in an HGSOC mouse model [14]. In addition, we evaluated four known SHetA2 drug targets. Three of the twenty proteins, ALDH1A3, TPI1, and TPM1, were elevated in ascites specimens that were sensitive to SHetA2. These three proteins were considered to be identified, but not yet validated, as candidate biomarkers for predicting SHetA2 sensitivity in patients with recurrent ovarian cancer.

The specificity of our approach for the identification of SHetA2 biomarkers was corroborated by employing the same methodology and samples categorized as sensitive or resistant under olaparib treatment. There were no significant correlations between SHetA2 and olaparib potencies, and a unique biomarker pattern was identified for olaparib response with higher levels of MAPK1, TPI1 or TPM1 being associated with olaparib resistance. The increased expression of MAPK1 in samples resistant to olaparib is consistent with the established association between the RAS/MAPK pathway and resistance to PARP inhibitors [42,43,44]. The findings in our study are consistent with other recent data suggesting that targeting the RAS/MAPK pathway could resensitize resistant cancer cells to PARP inhibitors [43,45]. The effectiveness of the combined administration of olaparib and a RAS/MAPK inhibitor in patients with epithelial ovarian cancer resistant to PARP inhibitors is currently under investigation in an ongoing phase I/II clinical trial (clinicaltrials.gov: NCT03162627) [42].

TPI1 exhibited opposite patterns of association with sensitivity and resistance for SHetA2 and olaparib and remained significant when *p* values were corrected for multiple comparisons. The association of elevated TPI1 with olaparib resistance and SHetA2 sensitivity within the same set of specimens suggests that TPI1 could be a biomarker for which patients with olaparib-resistant HGSOC could benefit from SHetA2 treatment. Furthermore, the pattern of SHetA2 versus olaparib sensitivities, with most of the cultures being resistant to olaparib while being sensitive to SHetA2, supports the development of SHetA2 for treatment of patients with olaparib-resistant HGSOC.

Multiple types of validation are needed before a predictive biomarker can be utilized in clinical trials. We evaluated the functional roles of the TPI1 identified candidate biomarker in the mechanism of SHetA2 inhibition of metabolic viability. Our approach involved testing the effects of altering TPI1 levels on SHetA2 potencies using siRNA reduction, which significantly reduced SHetA2 potencies, and lentiviral vector-mediated overexpression, which significantly increased SHetA2 potencies in both HGSOC cell lines tested.

TPI1 catalyzes the fifth step of glycolysis involving the interconversion of dihydroxyacetone phosphate and glyceraldehyde-3-phosphate [46]. Inhibition of TPI1 reduced SHetA2 potency, whereas overexpression of TPI1 increased SHetA2 potency, suggesting that TPI1 is an active downstream effector of the mechanism by which SHetA2 reduces ovarian cancer metabolic viability. As TPI1 is a key metabolic enzyme involved in glycolysis, we evaluated SHetA2 treatment effects on glycolysis metabolites for further mechanistic validation and found that SHetA2 reduced glycolysis metabolites downstream of TPI1 and increased metabolites upstream of TPI1.

The role of TPI1 in SHetA2 sensitivity and reduction in its downstream glycolysis metabolites in SHetA2-treated HGSOC cells documents a role for inhibition of glycolysis in the SHetA2 anti-HGSOC mechanism. In support of this strategy, inhibition of hexokinase 2, which catalyzes the first step of glycolysis, reduced stem cell line properties in ovarian cancer cell lines [47]. Furthermore, inhibition of pyruvate dehydrogenase kinase 4 (PDK4) leading to reduction in glycolysis metabolites feeding into the TCA cycle was shown to inhibit stem cell-like properties in CSCs derived from ascites collected from patients with ovarian cancer [17]. A limitation of this treatment strategy is that inhibition of glycolysis is clearly not enough to prevent HGSOC recurrence based on the metabolic plasticity of HGSOC CSCs and their ability to continue utilizing oxidative phosphorylation/TCA cycle and the PPP in hostile microenvironments [19,48]. A promising characteristic of SHetA2 that may overcome this limitation is its ability to block oxidative phosphorylation in ovarian cancer cells and induce mitophagy that contributes to the mechanism of cell death [28]. Therefore, the dual inhibition of glycolysis and TCA cycle/oxidative phosphorylation in HGSOC cells by SHetA2 could prevent the metabolic plasticity of HGSOC from supporting drug resistance. SHetA2 could counteract both the upregulation of glycolysis in HGSOC cells and TCA cycle/oxidative phosphorylation in HGSOC CSCs as survival mechanisms in toxic microenvironments [17,18,19].

Drugs that inhibit oxidative phosphorylation have been considered a rational approach for targeting cancer stem cells [49]; however, their clinical utility may be limited by toxicity [50]. An initial example is IACS-010759, an inhibitor of mitochondrial complex 1, which demonstrated dose-limiting toxicities, a narrow therapeutic index and compensatory glycolysis in phase 1 trials of patients with advanced solid tumors or acute myeloid leukemia [51].

SHetA2 offers an alternative to direct inhibition of oxidative phosphorylation by instead disrupting the HSP70 chaperone protection of mitochondrial proteins and metabolic enzymes [28]. Non-cancer ovarian and fallopian tube epithelial cells overcome SHetA2 inhibition of oxidative phosphorylation through mitochondrial fusion and low-level autophagy, while ovarian cancer cells are deficient in these survival mechanisms [28]. To date, the ongoing phase 1 trial of oral SHetA2 capsules in patients with advanced and recurrent solid tumors is consistent with this differential toxicity between cancer and non-cancer cells with no occurrence of dose-limiting toxicities. Similarly to the observation for IACS-010759, compensatory glycolysis has been observed for SHetA2-treated high-risk human papillomavirus positive (HR-HPV+) cervical cancer cells [52]. However, compensatory glycolysis has not been observed in HR-HPV negative ovarian or endometrial cancer cells [28]. The difference in compensatory glycolysis in response to SHetA2 treatment between HR-HPV+- and non-HR-HPV-associated cancers is consistent with our observation that the presence of HR-HPV alters cancer cell survival responses to SHetA2 [53].

Overall, our metabolomic analysis demonstrated that SHetA2 inhibits multiple metabolic pathways in the ovarian cancer cells including energy- and building block-producing pathways (glycolysis, aspartate-malate shuttle, TCA cycle, creatine kinase pathway) and redox-quenching (oxidative PPP production of NADPH) pathways in the cells. The reduction in oxidative PPP and significant increase in NADP+ may explain the significant reduction in proline in SHetA2-treated cells. Elevated aromatic amino acids and some nucleosides and nucleotides could be caused by the upregulation of the non-oxidative PPP in SHetA2-treated cells and also by the known SHetA2 induction of autophagy in ovarian cancer cells [28]. Currently, it is unclear why SHetA2 represses oxidative PPP and increases non-oxidative PPP in ovarian cancer cells; however, it is interesting to note that the increase in non-oxidative PPP is fed by glycolysis metabolites that could accumulate upstream of SHetA2 inhibition of TPI1.

Although our evidence supports the glycolysis activity of TPI1 in the SHetA2 mechanism, this protein has several moonlighting functions that may also contribute to or possibly interfere with the anti-cancer activity of SHetA2 [54]. Oxidative stress or chemotherapy can induce TPI1 phosphorylation at serine 80 by cyclin-dependent kinase 2 (CDK2), leading to nuclear localization of TPI1 in lung cancer cells where it confers chemoresistance through a non-catalytic activity [55]. In the nucleus, TPI1 can acetylate histones through a mechanism dependent upon its isomerase activity [56]. Other nuclear activities of TPI1 appear to be independent of its isomerase activity [55]. Multiple kinases interact with TPI1 to promote cell proliferation and migration, including mammalian target of rapamycin complex 1 (mTORC1) upstream of CDK2 and cell division cycle associated 5 (CDCA5) upstream of phosphatidylinositol-3-kinase (PI3K) and protein kinase B (Akt/PKB) [55].

The other two identified biomarkers of SHetA2 sensitivity, ALDH1A3 and TPM1, did not pass the stringency of validation in this study; however, their association with SHetA2 and ovarian cancer warrants further study. Their correlation with SHetA2 sensitivity may be clinically useful if it predicts drug sensitivity even if the proteins are not directly involved in the drug mechanism of action. Studies with larger numbers of samples may provide sufficient power to prove their involvement in the SHetA2 mechanism.

Although SHetA2 has been shown to bind the three HSP70 family members, Grp78, hsc70 and mortalin [26,28], and cause degradation of cyclin D1 [25], the expression levels of these proteins were not associated with SHetA2 sensitivity in this study. Because SHetA2 disrupts the function of the HSP70 proteins without decreasing their expression levels or causing their degradation [28], this observed lack of association between expression levels and drug sensitivity was not unexpected. The lack of association between cyclin D1 levels and SHetA2 sensitivity was not predicted, however, and may be explained by the cell death mechanisms involving mitochondrial damage having superiority over the cell cycle-regulating mechanisms involving cyclin D1 in mediating SHetA2 sensitivity.

The limitations of this study include the small sample size of patients with HGSOC and the heterogenous nature of ascites specimens. Additional studies of TPI1 expression using independent sets of SHetA2-treated and control ascites-derived cultures, blood and tumor tissues are needed to support the validity of TPI1 as a predictive SHetA2 sensitivity biomarker. Further mechanistic studies of how TPI1 mediates SHetA2 anti-cancer effects are needed to verify whether the effects involve the metabolic activities of TPI1 exclusively or additional moonlighting functions of TPI1. It remains to be determined whether SHetA2 directly binds to TPI1 or indirectly affects its function through disrupting its HSP70 chaperone protection.

## 5. Conclusions

ALDH1A3, TPI1, and TPM1 expression levels were associated with SHetA2 sensitivity and MAPK1, TPI1 and TMP1 expression levels were associated with olaparib resistance in HGSOC ascites-derived cultures. The specificity of these candidate biomarkers for drug sensitivity was supported by the non-overlapping profiles of predictive biomarkers identified for SHetA2 and olaparib, with TPI1 and TPM1 expression having opposing directions for predicting SHetA2 versus olaparib sensitivities. The majority of specimens were resistant to olaparib and sensitive to SHetA2, supporting the development of SHetA2 as an alternative therapy for patients who develop resistance or intolerance to olaparib or other PARP inhibitors. The TPI1 glycolytic enzyme exhibited the strongest level of validation as a biomarker of SHetA2 sensitivity and is implicated in the metabolic mechanism by which SHetA2 reduces the viability of ovarian cancer cells.

## Figures and Tables

**Table 1:** SHetA2 and olaparib sensitivities of ascites-derived cultures.

Sample Name	Previous Patient Treatment	SHetA2 IC_50_ (μM)	SHetA2 Category	Olaparib IC_50_ (μM)	Olaparib Category	Used in Discovery Analysis	Used in Specificity Analysis	Used in Correlation Analysis
ASC 4	Carboplatin	UD	R	UD	R	√	√	√
ASC 7	RM 720	UD	R	2.6	S	√	√	√
ASC 12	RM 720	4.9	S	3	S	√	√	√
ASC 21	RM 720	7.0	S	10.4	R	√	√	√
ASC 23	RM 720	18.3	S	31.2	R	√	√	√
ASC 24	IMGN151	0.8	S	41.8	R	√	√	√
ASC 25	Carboplatin	UD	R	4.6	S	√	√	-
ASC 32	Carboplatin/Paclitaxel/Bevacizumab	10.4	S	20.1	R	-	-	√
ASC 62	Paclitaxel/Bevacizumab	5.3	S	10.1	R	-	-	√

UD: undefined. R: Resistant. S: Sensitive. √: Used in specified analysis. -: Not used in specified analysis.

**Table 2:** Numbers of ascites cultures sensitive or resistant to SHetA2 or olaparib.

	SHetA2 Sensitive	SHetA2 Resistant
Olaparib-Sensitive	1	2
Olaparib-Resistant	5	1

**Table 3:** Effects of TPI1 reduction on SHetA2 IC_50_ values in comparison to experimental controls.

Experiment	Replicate 1			Replicate 2			Replicate 3		
	Scrambled siRNA IC_50_	TPI-1 siRNA IC_50_	*p*-value *	Scrambled siRNA IC_50_	TPI-1 siRNA IC_50_	*p*-value	Scrambled siRNA IC_50_	TPI-1 siRNA IC_50_	*p*-value
ES-2 siRNA	4.31	5.18	<0.0001	2.68	4.98	<0.0001	4.47	4.92	<0.0001
MESOV siRNA	5.66	68.28	0.0002	5.75	6.69	0.0374	8.16	11.09	0.0163
	Empty vector IC_50_	TPI-1 vector IC_50_	*p*-value	Empty vector IC_50_	TPI-1 vector IC_50_	*p*-value	Empty vector IC_50_	TPI-1 vector IC_50_	*p*-value
ES-2 OE ^a^	8.17	4.43	<0.0001	7.55	1.87	<0.0001	4.92	4.64	0.913 ^b^
MESOV OE	6.16	4.77	<0.001	7.41	4.67	0.003	5.70	5.06	<0.001

* Comparison of curve fits the extra sum-of-squares F test. ^a^ OE = Overexpression. ^b^ TPI OE = only 1.03-fold.

## Data Availability

The original contributions presented in this study are included in the article/Supplementary Material. Further inquiries can be directed to the corresponding author.

## Supplementary Materials

The following supporting information can be downloaded at https://www.mdpi.com/article/10.3390/cells15030267/s1, Supplementary Table S1: Antibodies, dilutions and *p*-values for *t*-tests comparing protein expression levels between drug sensitivity groups; Supplementary Table S2: Raw Metabolomics Data of ES-2 cells treated with 5 micromolar SHetA2; Supplementary Table S3: Metabolites Significantly Altered by 5 min of SHetA2 Treatment; Supplementary Table S4: Metabolites Significantly Altered by 60 Minutes of SHetA2 Treatment.

## Abbreviations

The following abbreviations are used in this manuscript:

ALDH1A3	Aldehyde Dehydrogenase 1 Family Member A3
ATP	Adenosine Triphosphate
AUC	Area Under the Curve
BCA	Bicinchoninic Acid
CDK2	Cyclin-Dependent Kinase 2
CSCs	Cancer Stem Cells
DMEM	Dulbecco’s Modified Eagle Medium
DMSO	Dimethyl Sulfoxide
FBS	Fetal Bovine Serum
G3P	Glycerol-3-Phosphate
Grp78	Glucose-Regulated Protein 78
HGSOC	High-Grade Serous Ovarian Carcinoma
HSP70	Heat Shock Protein 70 kDa family
hsc70	Heat Shock Cognate 70
IC_25_	25% Inhibitory Concentration
IC_50_	50% Inhibitory Concentration
IRB	Institutional Review Board
LDHA	Lactate Dehydrogenase
MAPK1	Mitogen-activated Protein Kinase 1
MES-OV	Human Ovarian Cancer Cell Line MES-OV
Mitophagy	Mitochondria-selective autophagy
NME1	Nucleoside Diphosphate Kinase 1
OE	Overexpression
OU	University of Oklahoma
PARP	Poly (ADP-Ribose) Polymerase
PDK	Pyruvate Dehydrogenase Kinase
rpm	Revolutions Per Minute
RRID	Research Resource Identifier
SCC	Stephenson Cancer Center
siRNA	Small Interfering RNA
STIC	Serous Tubal Intraepithelial Carcinoma
TCA cycle	Tricarboxylic Acid Cycle
TPM1	Tropomyosin 1
T-PER	Tissue Protein Extraction Reagent
TPI1	Triosephosphate Isomerase 1
UD	Undefined
